# Feasibility of a field‐based submaximal stair ascent test for estimating aerobic capacity and lower‐limb strength in middle‐aged and older adults

**DOI:** 10.1111/cpf.70073

**Published:** 2026-06-05

**Authors:** Matti Hyvärinen, Niina Kajan, Eeva Kettunen, Ari Lehtiö, Jouni Kallio, Keegan Knittle, Neil Cronin, Laura Karavirta

**Affiliations:** ^1^ Gerontology Research Center, Faculty of Sport and Health Sciences University of Jyväskylä Jyväskylä Finland; ^2^ Digital Services, University of Jyväskylä Jyväskylä Finland; ^3^ City of Jyväskylä Jyväskylä Finland; ^4^ Faculty of Sport and Health Sciences University of Jyväskylä Jyväskylä Finland; ^5^ School of Education and Science University of Gloucestershire, The Park Cheltenham UK

**Keywords:** cardiorespiratory fitness, field testing, physical performance, stair climbing, submaximal exercise test

## Abstract

**Background:**

Assessing functional capacity is a key aspect of exercise testing and offers important insights into health in adult populations. However, accurate assessment often requires laboratory‐based maximal tests. This pilot study investigated whether a field‐based self‐paced submaximal stair ascent test could feasibly assess aerobic capacity and lower‐limb strength in middle‐aged and older adults.

**Methods:**

Twenty‐eight participants (20 women, 8 men) aged 40 to 70 years completed maximal treadmill and isometric leg press tests to assess maximal oxygen consumption (VO_2_max) and lower‐limb strength, respectively. Participants also performed two separate self‐paced stair ascents of four and six flights. Ascent time, mean vertical power, as well as rate of perceived exertion, absolute heart rate, and relative heart rate following the test were recorded.

**Results:**

Ascent time and mean vertical power were associated with VO_2_max and lower‐limb strength, with standardized regression coefficients ranging from −0.61 to −0.27 and from 0.41 to 0.73, respectively. However, these associations were attenuated and no longer statistically significant after controlling for age and sex. Predictive models based on the two best‐performing stair ascent predictors selected by LASSO regression showed weak‐to‐moderate ability to predict VO_2_max (*R* ^2^ = 0.21–0.52) with cross‐validated model performance significantly different from zero. In contrast, the models did not significantly predict lower‐limb strength.

**Conclusions:**

These preliminary findings suggest that a brief self‐paced stair ascent test may offer a feasible field‐based approach for estimating aerobic capacity in middle‐aged and older adults. However, its predictive accuracy appears limited and should be further examined in larger samples.

## 1 INTRODUCTION

Functional capacity is a multidimensional construct reflecting individual's ability to perform activities of daily living and maintain independence. Aerobic capacity is one of its key physiological determinants and has consistently been associated with several important public health outcomes. For instance, greater aerobic capacity, often assessed as maximal oxygen consumption (VO2max), has been associated with decreased risk of cardiovascular diseases, type II diabetes, and mortality (Kodama, 2009; Lang et al., 2024), and with higher levels of physical activity (Hyvärinen et al., 2025). In addition to aerobic capacity, neuromuscular factors, such as lower‐limb strength, play an important role in physically demanding daily activities that involve supporting and repeatedly lifting body mass against gravity (Guralnik et al., 2000).

Despite the established importance of aerobic capacity and neuromuscular function for health and independent living, their accurate assessment often requires laboratory‐based maximal tests. Such tests are often impractical for large‐scale studies and health evaluations, especially among individuals with reduced physical and functional capacity (Sartor et al., 2013). This has increased the demand for accessible, submaximal methods that can be safely applied across individuals with varying levels of functional capacity. Ideally, these methods would require minimal equipment and allow independent testing, enabling individuals to monitor their functional capacity and health outside laboratory or clinical setting.

To address this need, several field‐based tests for assessing aerobic capacity have been developed, most commonly involving running (Cooper, 1968; Léger et al., 1988) or walking (Enright, 2003; Mänttäri et al., 2018) on a level surface. However, running‐based tests may not be suitable for all individuals due to their high mechanical and cardiovascular demands, increasing the risk of musculoskeletal injury and adverse cardiovascular events (American College of Sports Medicine, 2021; Fletcher et al., 2013). Conversely, walking tests conducted on level surfaces may be insufficiently demanding for individuals with higher functional capacity, potentially leading to ceiling effects and reduced sensitivity in the assessment of aerobic capacity (Halliday et al., 2020; Ross et al., 2010).

One less studied approach for assessing functional capacity is stair ascent, a common form of physical activity in everyday life. public places. Stair ascent requires the coordinated integration of cardiorespiratory fitness, lower‐limb strength and neuromuscular control (McFadyen & Winter, 1988; Teh & Aziz, 2002). Compared with level walking, stair ascent involves repeated lifting of body mass against gravity, resulting in substantially higher metabolic and mechanical demands (Ainsworth et al., 2011; Lin et al., 2015). Furthermore, limitations in stair‐climbing performance have been consistently associated with reduced physical functioning and increased risk of disability in middle‐aged and older adults (Lange‐Maia et al., 2019; Tiedemann et al., 2007).

Movement patterns similar to stair ascent have field‐based step tests also been previously used in the context of submaximal field‐based testing with different step tests that incorporate stepping onto and off a step platform (Åstrand & Ryhming, 1954; Siconolfi et al., 1982). However, these tests often involve step platforms that are higher than typical stairs and require stepping backwards, a movement less common in daily life and potentially challenging for individuals with reduced functional capacity. In contrast, self‐paced stair ascent closely reflects real‐world movement demands and may represent a safe and promising approach for assessing functional capacity in middle‐aged and older adults. Due to its relatively high mechanical demands, stair ascent tests may also provide insight into lower‐limb strength in addition to aerobic capacity.

Thus, the aim of this study was to examine the potential of a self‐paced submaximal stair ascent test for assessing functional capacity. Specifically, we investigated the associations of stair ascent measures with aerobic capacity and lower‐limb strength and evaluated the feasibility of prediction models to estimate these outcomes in middle‐aged and older adults.

## 2 METHODS

### 2.1 Participant recruitment and study design

The study targeted healthy, sedentary men and women aged 40–70 years. The participants were recruited with announcements on bulletin boards at nearby workplaces and on social media channels in the Jyväskylä region. A total of 38 participants expressed interest in the study by completing an electronic pre‐screening survey, which was used to ensure that participants were eligible for the study. Of the 38 individuals screened, seven did not meet the inclusion criteria, one was unable to attend the laboratory measurements, and two were excluded based on the study physician's assessment during the first laboratory visit. The final study sample, therefore, consisted of 28 participants (20 women and 8 men).

The exclusion criteria included chronic diseases requiring medication, use of beta‐blockers or other heart rate‐affecting medications, severe musculoskeletal conditions that could interfere with the laboratory tests, body mass index (BMI) greater than 30 kg·m^−^^2^, engagement in regular moderate‐to‐vigorous physical activity for more than 60 min per week, smoking or use of other nicotine products, pregnancy, and night‐shift work. Engagement in regular physical activity was assessed using an eight‐level self‐report scale (0–7), where 0 indicated “I avoid walking or exercise” and 7 indicated “I participate regularly in heavy physical exercise, such as running 15 km per week or engaging in more than 3 h per week of comparable physical activity” (Jackson et al., 1990). The aim was to recruit sedentary participants with an activity level of 2 or lower; however, some participants with higher values (i.e., 3 or 5) were included due to seasonal variation in their physical activity.

The study protocol included two laboratory visits scheduled approximately 1 week apart, with a minimum of 7 and a maximum of 11 days between the visits. During the first visit, participants completed self‐report questionnaires, including questions on age, sex, health, and lifestyle, and underwent assessments of body composition and functional capacity. Stair ascent tests were performed during the second visit. All visits took place between January and March 2025. The study was approved by the Human Sciences Ethics Committee of the University of Jyväskylä, and all participants provided written informed consent prior to participation. The study followed the principles outlined by the Declaration of Helsinki.

### 2.2 Functional capacity and anthropometrics

Lower‐limb strength was assessed using a maximal bilateral isometric leg press test using a custom‐built dynamometer (Häkkinen et al., 1998). The test was performed in a seated position with a knee angle of 107°. Participants were instructed to generate maximum force against the plate for approximately 3 s, and the momentary maximal value was recorded. After three submaximal warm‐up trials, participants performed a minimum of three maximal trials, with a 1‐min break in between the contractions. If the result of the last trial exceeded the previous maximum by more than 5%, an additional trial was performed, with a maximum of five trials in total. The highest peak force achieved was divided by body mass and used as the measure of lower‐limb strength in the analysis.

Aerobic capacity was assessed by measuring VO_2_max using a modified Balke protocol on a treadmill (h/p/cosmos Quasar Med, Nussdorf‐Traunstein, Germany) (Aadland et al., 2017; Balke & Ware, 1959). Briefly, the protocol began with a 3‐min familiarization period consisting of walking on a level treadmill at progressively increasing speeds, reaching a starting speed of 3.8 km/h for participants aged over 55 years and 4.8 km/h for participants aged under 55 years. The treadmill was then set to a 4% inclination, which was increased by 2% each minute until a maximum incline of 20% was reached. For participants who continued beyond 12 min, the treadmill speed was subsequently increased by 0.5 km/h per minute until volitional exhaustion. Oxygen consumption was measured using a Vyntus CPX gas analyzer (CareFusion GmbH). Volume and gas calibrations were performed before each test according to the manufacturer's recommendations. A test was considered maximal if the participant achieved a respiratory exchange ratio greater than 1.10 or a rate of perceived exertion (RPE) greater than 17 on the Borg 6–20 scale (Borg, 1970).

Prior to the VO_2_max test, all participants were classified as either low or moderate risk based on the American College of Sports Medicine risk stratification guidelines (American College of Sports Medicine, 2021). For all participants classified as moderate risk (*n* = 21), the VO_2_max test was supervised by a physician. For example, all sedentary men aged over 45 and all women over 55 were classified as moderate risk participants.

Absolute VO_2_max (mL·min^−1^), defined as the highest binned 30‐second mean oxygen uptake, was divided by body mass to calculate VO_2_max relative to body mass (mL·min^−1^·kg^−1^). Although VO_2_max relative to body mass is commonly used in practice and in the literature, this approach has been shown to overestimate the influence of body mass on aerobic capacity (Nevill et al., 1992). Therefore, we also used allometrically scaled VO_2_max (mL·min^−1^·kg^−2/3^), which was calculated by dividing absolute VO_2_max by total body mass raised to the power of two‐thirds. Body mass and body height were measured using the InBody 970 and its built‐in stadiometer (InBody Co. Ltd.).

### 2.3 Self‐paced stair ascents

The stair ascents were conducted in the same indoor staircases, separately for ascents of four and six flights. Each flight consisted of 19–22 steps, with one or two short horizontal transitions within each flight. The four‐flight ascent included 81 steps with a total vertical displacement of 14.28 m. The six‐flight ascent included 121 steps with a total vertical displacement of 21.48 m.

Participants were instructed to perform each ascent by walking at a constant, self‐selected brisk submaximal pace. During the ascents, participants wore a next‐generation inertial measurement unit (NGIMU) device (x‐io Technologies Limited) attached to their lower back and a Polar H10 heart rate sensor (Polar Electro Oy, Kempele, Finland) attached to the chest using elastic straps. Immediately after each ascent, participants were asked to report their RPE on the Borg 6–20 scale (Borg, 1970), and heart rate from the Polar H10 was recorded using the Kubios HRV mobile application (Kubios Oy; version 1.6.3). Ascent time was measured using a stopwatch and verified using barometric pressure and three‐axis acceleration derived from the NGIMU measurements with custom‐written MATLAB code (MathWorks Inc, 2024). The code used barometric pressure to detect the timing of the ascent, while the exact test duration was determined by step detection using a threshold‐crossing algorithm applied to the filtered resultant acceleration signal.

Mean vertical power (W) was computed by dividing the product of body mass (kg), total vertical displacement of the ascent (m), and gravitational acceleration (9.807 m·s^−2^) by ascent time (s). In addition to absolute post‐ascent heart rate, heart rate relative to age‐predicted maximum (Tanaka et al., 2001) was also included in the analyses, and calculated by dividing the post‐ascent heart rate by 208 – 0.7·age.

### 2.4 Statistical analysis

The associations between stair ascent measures and functional capacity were investigated using linear regression models. Standardized regression coefficients and their 95% confidence intervals were derived from both simple linear regression (unadjusted models) and multiple linear regression models adjusted for age and sex. All models were constructed separately for each stair ascent measure and outcome variable. Outcome variables included VO_2_max normalized to body mass, allometrically normalized VO_2_max, and maximal isometric leg press normalized to body mass. Residual plots, Q–Q plots, and correlation coefficients were used to ensure that there were no significant violations of the model assumptions. All analyses were carried out in R (R Core Team, 2025).

To identify the most relevant predictors of functional capacity from stair ascent measures, the best set of predictors was chosen using least absolute shrinkage and selection operator (LASSO) regression (Tibshirani, 1996) implemented in R with the ‘glmnet’ package (Pinheiro J, Bates D, DebRoy S, Sarkar D, R Core Team, n.d.). Due to the relatively small sample size and the primary focus on stair ascent measures, participant characteristics were not included in the set of potential predictors. Furthermore, to reduce the risk of overfitting given the limited sample size, the number of predictors in the final models was limited to two by tuning the LASSO regularization parameter (λ). Depending on the number of ascended flights and the outcome variable, the best‐performing pair of predictors consisted of either mean vertical power and RPE or mean vertical power and relative heart rate. Therefore, the subsequent analyses were conducted separately using both predictor pairs.

Model performance was first evaluated using the coefficient of determination (*R* ^2^) with 95% confidence intervals, as well as mean absolute error of the residuals and their standard deviations in the fitted models, to describe apparent (in‐sample) performance and maximum explanatory potential of the model. Given the small sample size, the primary models were restricted to two predictors; age and sex were added in subsequent models as part of a hierarchical approach to provide a sensitivity analysis. To assess predictive performance and reduce the optimism bias, leave‐one‐out cross‐validation (LOOCV) was applied. In addition, in order to improve the robustness of performance given the relatively small sample size, bootstrapping with 1000 resamples was used to estimate *R* ^2^ for the LOOCV models using the ‘boot’ package in R (Canty & Ripley, 2025).

## 3 RESULTS

### 3.1 Study population and stair ascents

The age range of the participants was 40 to 70 years, with a mean age of 54 years. Women were slightly older than men (56 vs. 49; Table 1). On average, the participants were slightly overweight, with a mean BMI of 26.3 kg·m^−2^ for both men and women. The participants demonstrated relatively good aerobic capacity, with mean VO_2_max values of 41 mL·min^−1^·kg^−1^ for men and 29 mL·min^−1^·kg^−1^ for women. The average self‐paced stair ascent test duration was 57 s for four‐flight ascent and 94 s for six‐flight ascent. For both ascents, men chose a faster ascending pace, as the mean ascent times for four‐ and six‐flight ascents were respectively 46 and 73 s for men and 62 and 103 s for women. The mean RPE and absolute heart rate values after the test were 11.5 and 134 bpm for the four‐flight ascent and 14.5 and 138 bpm for the six‐flight ascent, respectively.

**Table 1:** Participant and stair ascent characteristics.

	All participants (*n* = 28)	Men (*n* = 8)	Women (*n* = 20)
Participant characteristics			
Age [y]	54.3 ± 9.6	49.3 ± 7.6	56.3 ± 9.7
Body mass [kg]	75.2 ± 10.5	85.4 ± 11.0	71.1 ± 7.1
Height [m]	1.69 ± 0.84	1.80 ± 0.37	1.65 ± 0.48
Body mass index [kg·m^−2^]	26.3 ± 3.1	26.3 ± 3.4	26.3 ± 3.0
Self‐reported physical activity level^a^	2 (1–3)	3 (1–5)	2 (1–3)
VO_2_max normalized to body mass [mL·kg^−1^·min^−1^]	32.9 ± 6.8	41.2 ± 4.5	29.2 ± 4.2
Allometrically normalized VO_2_max [mL·kg^−1·^min^−2/3^]	139 ± 32	181 ± 18	122 ± 16
Isometric leg press normalized to body mass [N·kg^−1^]	33.6 ± 9.4	39.6 ± 7.6	31.3 ± 9.2
Self‐paced stair ascent characteristics			
Four‐flight stair ascent			
Ascent time [s]	57.6 ± 12.7	46.4 ± 5.6	62.2 ± 11.9
Mean vertical power [W]	193 ± 59	262 ± 46	166 ± 37
RPE (post)	11.5 ± 2.5	10.8 ± 2.1	11.8 ± 2.6
Heart rate (post) [bpm]	134 ± 16	130 ± 18	136 ± 16
Relative heart rate [%]	79.2 ± 9.5	74.8 ± 9.2	81.0 ± 9.3
Six‐flight stair ascent			
Ascent time [s]	94.3 ± 22.4	72.6 ± 9.3	103.1 ± 20.0
Mean vertical power [W]	180 ± 61	253 ± 50	151 ± 35
RPE	14.5 ± 1.9	13.5 ± 2.0	15.0 ± 1.8
Heart rate [bpm]	138 ± 19	137 ± 18	139 ± 20
Relative heart rate [%]	81.6 ± 11.1	79.2 ± 9.4	82.6 ± 11.8

*Note*: Data are mean ± standard deviation unless otherwise specified.

Abbreviations: RPE, rate of perceived exertion; VO_2_max, maximal oxygen consumption.

^a^ Data are median (interquartile range).

### 3.2 Associations between stair ascent measures and functional capacity

In the unadjusted models, shorter ascent time and greater mean vertical power were associated with better functional capacity across all outcome variables, regardless of test length (Table 2). For ascent time and mean vertical power, the standardized regression coefficients (*β*) ranged from −0.61 to −0.27 and from 0.41 to 0.73, respectively, across all outcome variables and both stair ascent tests. RPE, absolute heart rate, and relative heart rate after the test were not significantly associated with the functional capacity outcome variables. However, the effect sizes were stronger for RPE and relative heart rate than for absolute heart rate, with *β* values ranging from −0.36 to −0.17 for RPE and relative heart rate.

**Table 2:** Associations between stair ascent measures and functional capacity (*n* = 28).

	VO_2_ max normalized to body mass		Allometrically normalized VO_2_max		Isometric leg press normalized to body mass	
	*β*	95% CI	*β*	95% CI	*β*	95% CI
Unadjusted models						
Four‐flight stair ascent						
Ascent time	−0.523**	(−0.867, −0.180)	−0.589***	(−0.915, −0.263)	−0.276	(−0.663, 0.111)
Mean vertical power	0.595***	(0.271, 0.919)	0.725***	(0.447, 1.000)	0.414*	(0.047, 0.781)
RPE	−0.109	(−0.510, 0.292)	−0.111	(−0.511, 0.290)	−0.276	(−0.663, 0.111)
Heart rate	−0.088	(−0.489, 0.314)	−0.104	(−0.505, 0.297)	−0.159	(−0.557, 0.239)
Relative heart rate	−0.244	(−0.635, 0.146)	−0.264	(−0.653, 0.125)	−0.341	(−0.720, 0.038)
Six‐flight stair ascent						
Ascent time	−0.549**	(−0.886, −0.212)	−0.606***	(−0.926, −0.285)	−0.425*	(−0.790, −0.060)
Mean vertical power	0.595***	(0.271, 0.919)	0.719***	(0.439, 0.999)	0.465*	(0.108, 0.822)
RPE	−0.358	(−0.734, 0.019)	−0.339	(−0.718, 0.040)	−0.318	(−0.700, 0.065)
Heart rate	−0.038	(−0.441, 0.365)	−0.212	(−0.424, 0.382)	−0.136	(−0.535, 0.263)
Relative heart rate	−0.179	(−0.575, 0.218)	−0.164	(−0.561, 0.234)	−0.293	(−0.678, 0.093)
Models adjusted for age and sex						
Four‐flight stair ascent						
Ascent time	−0.140	(−0.429, 0.149)	−0.179	(−0.413, 0.054)	−0.135	(−0.541, 0.271)
Mean vertical power	0.013	(−0.351, 0.377)	0.198	(−0.094, 0.490)	0.278	(−0.214, 0.770)
RPE	0.026	(−0.218, 0.270)	0.037	(−0.166, 0.241)	−0.234	(−0.559, 0.092)
Heart rate	−0.010	(−0.263, 0.244)	−0.009	(−0.220, 0.203)	−0.237	(−0.575, 0.101)
Relative heart rate	−0.005	(−0.257, 0.246)	−0.006	(−0.215, 0.204)	−0.228	(−0.564, 0.108)
Six‐flight stair ascent						
Ascent time	−0.142	(−0.449, 0.165)	−0.164	(−0.416, 0.087)	−0.397	(−0.798, 0.004)
Mean vertical power	0.007	(−0.373, 0.387)	0.190	(−0.117, 0.496)	0.475	(−0.014, 0.963)
RPE	−0.049	(−0.310, 0.211)	−0.005	(−0.223, 0.213)	−0.107	(−0.467, 0.254)
Heart rate	−0.052	(−0.295, 0.191)	−0.029	(−0.232, 0.174)	−0.210	(−0.537, 0.118)
Relative heart rate	−0.051	(−0.293, 0.192)	−0.027	(−0.230, 0.175)	−0.203	(−0.530, 0.125)

Abbreviations: *β*, standardized regression coefficient; CI, confidence interval; RPE, rate of perceived exertion; VO_2_max, maximal oxygen consumption.

*** *p* < 0.001

** *p* < 0.01

* *p* < 0.05.

After adjustment for age and sex, the effect sizes were clearly weaker and no longer statistically significant, with *β* values ranging from −0.40 to −0.13 for ascent time, from 0.00 to 0.48 for mean vertical power, and from −0.24 to 0.03 for RPE and relative heart rate (Table 2). Notably, the associations were consistently stronger with allometrically normalized VO_2_max than with VO_2_max normalized to body mass across all models.

### 3.3 Using stair ascent measures to estimate functional capacity

The apparent performance of the models, including mean vertical power and either RPE or relative heart rate was moderate, with *R* ^2^ values ranging from 0.23 to 0.60 across all outcome variables and both stair ascent tests (Table 3; Supplementary Tables 1–3). This indicates that these pairs of stair ascent measures explained approximately one‐quarter to nearly two‐thirds of the variance in the outcome variables in the present sample. Models including mean vertical power and RPE tended to show better apparent performance than those including mean vertical power and relative heart rate. Furthermore, stair ascent measures demonstrated better apparent performance for aerobic capacity outcomes, particularly allometrically scaled VO_2_max (*R* ^2^ = 0.53–0.60), than for lower‐limb strength assessed by isometric leg press normalized to body mass (*R* ^2^ = 0.23–0.29).

**Table 3:** Apparent performance of self‐paced stair ascent measures for estimating functional capacity outcomes (*n* = 28).

	VO_2_max normalized to body mass [mL·min^−1^·kg^−1^]	Allometrically normalized VO_2_max [mL·min^−1^·kg^−2/3^]	Isometric leg press normalized to body mass [N·kg^−1^]
Mean vertical power and RPE as predictors			
Four‐flight stair ascent			
*R* ^2^ (95% CI)	0.36 (0.13, 0.68)	0.54 (0.27, 0.81)	0.24 (0.11, 0.52)
Mean absolute error ± SD	4.16 ± 3.43	16.66 ± 13.54	6.53 ± 4.76
Six‐flight stair ascent			
*R* ^2^ (95% CI)	0.45 (0.20, 0.70)	0.60 (0.35, 0.82)	0.30 (0.12, 0.57)
Mean absolute error ± SD	3.86 ± 3.20	14.92 ± 13.31	6.23 ± 4.70
Mean vertical power and relative heart rate as predictors			
Four‐flight stair ascent			
*R* ^2^ (95% CI)	0.36 (0.11, 0.66)	0.53 (0.28, 0.79)	0.23 (0.09, 0.50)
Mean absolute error ± SD	4.33 ± 3.20	17.21 ± 12.91	6.97 ± 4.19
Six‐flight stair ascent			
R^2^ (95% CI)	0.38 (0.14, 0.67)	0.54 (0.29, 0.78)	0.29 (0.14, 0.56)
Mean absolute error ± SD	4.30 ± 3.13	17.09 ± 12.87	6.44 ± 4.40

Abbreviations: CI, confidence interval; *R* ^2^, coefficient of determination; SD, standard deviation; VO_2_max, maximal oxygen consumption. Highlighted *R* ^2^ values differ statistically significantly from zero.

When assessing the generalizability of the models using LOOCV, both predictor pairs demonstrated moderate predictive performance for allometrically scaled VO_2_max, with *R* ^2^ values ranging from 0.43 to 0.52 and mean absolute errors varying from 14.9 to 17.2 mL·min^−1^·kg^−2/3^ (Table 4). In contrast, predictive performance for VO_2_max normalized to body mass was weak to moderate, with *R* ^2^ values ranging from 0.21 to 0.34 and mean absolute errors varying from 3.8 to 4.4 mL·min^−1^·kg^−1^. Notably, only the model including mean vertical power and RPE showed an *R* ^2^ that was statistically different from zero (*R* ^2^ = 0.34, 95% confidence interval 0.03–0.53). None of the models demonstrated meaningful predictive performance for lower‐limb strength, as indicated by low and non‐significant *R* ^2^ values.

**Table 4:** Predictive performance of self‐paced stair ascent measures using leave‐one‐out cross‐validation (*n* = 28).

	VO_2_max normalized to body mass [mL·min^−1^·kg^−1^]	Allometrically normalized VO_2_max [mL·min^−1^·kg^−2/3^]	Isometric leg press normalized to body mass [N·kg^−1^]
Mean vertical power and RPE as predictors			
Four‐flight stair ascent			
*R* ^2^ (95% CI)	0.21 (−0.08, 0.61)	0.43 (0.06, 0.77)	0.08 (−0.10, 0.43)
Mean absolute error ± SD	4.65 ± 3.77	18.64 ± 14.80	7.24 ± 5.15
Six‐flight stair ascent			
*R* ^2^ (95% CI)	0.34 (0.03, 0.63)	0.52 (0.17, 0.79)	0.15 (−0.07, 0.47)
Mean absolute error ± SD	4.29 ± 3.46	16.49 ± 14.42	6.90 ± 5.05
Mean vertical power and relative heart rate as predictors			
Four‐flight stair ascent			
*R* ^2^ (95% CI)	0.24 (−0.08, 0.60)	0.43 (0.07, 0.76)	0.05 (−0.13, 0.38)
Mean absolute error ± SD	4.79 ± 3.45	19.09 ± 13.97	7.77 ± 4.58
Six‐flight stair ascent			
*R* ^2^ (95% CI)	0.26 (−0.05, 0.61)	0.45 (0.10, 0.76)	0.12 (−0.08, 0.45)
Mean absolute error ± SD	4.75 ± 3.32	19.00 ± 13.71	7.20 ± 4.86

Abbreviations: CI, confidence interval; *R* ^2^, coefficient of determination; SD, standard deviation; VO_2_max, maximal oxygen consumption. Highlighted *R* ^2^ values differ statistically significantly from zero.

## 4 DISCUSSION

In this pilot study of 28 middle‐aged and older adults, we observed that self‐paced stair ascent measures, particularly ascent time and mean vertical power, were associated with aerobic capacity and lower‐limb strength. However, these associations were no longer present after adjustment for age and sex, suggesting that the observed relationships may not be independent of these background characteristics, reflecting shared age‐ and sex‐related variation in aerobic capacity and lower‐limb strength in line with the established physiological determinants of these components. Furthermore, models including the best‐performing pairs of stair ascent predictors, that is, mean vertical power together with relative heart rate or RPE, demonstrated weak to moderate predictive performance for estimating aerobic capacity.

We observed that the pace participants chose for the stair ascents was associated with their aerobic capacity and lower‐limb strength. This finding is in agreement with previous literature, as participants with better functional capacity naturally carry out everyday tasks in a more demanding or vigorous manner, for example, during sit‐to‐stand transitions (Löppönen et al., 2022; Pickford et al., 2019). Furthermore, reduced physical functioning and increased risk of disability have previously been associated with limitations in stair‐ascending performance in adult populations (Lange‐Maia et al., 2019; Tiedemann et al., 2007).

However, the perceived and relative physiological intensity of the ascents, assessed using RPE and heart rate were not independently associated with the measures of functional capacity, either before or after adjustment for confounders. This finding may partly reflect the fact that, based on our instructions, participants tended to choose a similar relative intensity for stair ascent in relation to their individual fitness level. A further consideration is that the physiological response may be delayed during these relatively short ascents (under 2 min, even for the longer trials), especially given the high metabolic and mechanical demands of stair climbing (Ainsworth et al., 2011; Lin et al., 2015). Specifically, the validity of a single time‐point absolute or relative heart rate measurement for estimating physiological intensity may be limited, as achieving a steady‐state heart rate often requires a longer test duration, and there is substantial inter‐individual variation in heart rate dynamics at exercise onset (Bunc et al., 1988; Sietsema et al., 1989). This delayed physiological response, reflected in both heart rate and RPE kinetics, may partly explain the higher heart rate observed after six flights despite greater mean vertical power during the four‑flight ascent. It should be noted, however, that the longer ascent requires more work against gravity, which also explains the lower mean vertical power for six‐flight ascent. Furthermore, subjective ratings of RPE are inherently prone to bias due to individual differences in how exertion is interpreted. This variability may be even greater during stair ascent, as perceived effort may reflect either lower‐limb skeletal muscle fatigue or cardiorespiratory and metabolic strain (Halperin & Emanuel, 2020).

Nonetheless, feature selection using LASSO regression indicated that combining information on the perceived and relative physiological intensity of the ascents with self‐selected pace may provide additional value in assessing functional capacity. Including these measures allowed us to capture both activity‐based information via self‐selected pace, as well as subjective and physiological indicators of exertion via RPE and heart rate during the ascent. Consequently, our results suggest that combining the information from these self‐paced stair ascent measures can explain a meaningful proportion of the variance in aerobic capacity, particularly in our pilot study population, but likely also in other populations similar to our sample.

Specifically, our models performed best in predicting allometrically normalized VO_2_max, while also showing weaker but still meaningful predictive performance for VO_2_max normalized to body mass, with slightly better performance observed for models including the six‐flight stair ascent measures compared with the four‐flight ascent. Normalizing VO_2_max to body mass using ratio scaling has been shown to introduce systematic bias, tending to underestimate aerobic capacity in individuals with larger body mass and overestimate it in those with lower body mass (Krachler & Stovitz, 2019). This may explain the weaker predictive performance observed for VO_2_max normalized to body mass compared with allometrically normalized VO_2_max, as mean vertical power is directly proportional to body mass. Furthermore, these results indicate that a longer test duration may provide a more accurate estimate of aerobic capacity, which is plausible given that longer exercise bouts are more likely to reflect aerobic rather than anaerobic energy contribution (Gastin, 2001). In contrast, the stair ascent measures did not demonstrate an ability to accurately predict lower‐limb strength, as assessed by maximal isometric leg press normalized to body mass. This may reflect the fact that the submaximal stair ascent, involving approximately 40 to 60 repetitions per leg, represents a relatively low strength demand for healthy middle‐aged and older adults. However, in shorter or more intense ascents, or in populations with lower functional capacity, the contribution of muscular strength is likely to be more pronounced.

These results suggest that the self‐paced stair ascent with a vertical displacement of 14 to 22 m may be a feasible tool for predicting aerobic capacity, particularly when the influence of body mass is appropriately accounted for through allometric scaling (Nevill et al., 1992). Although mean vertical power had moderate‐to‐strong associations with both aerobic capacity and lower‐limb strength in the simple linear regression models, predictive models that also included RPE or relative heart rate showed moderate predictive performance only for aerobic capacity. This pattern suggests that, in our population, exertion captured by RPE and heart rate may reflect primarily cardiorespiratory and metabolic strain rather than lower‐limb skeletal muscle fatigue. This finding is consistent with previous literature, as heart rate and RPE are well‐established markers of cardiorespiratory and metabolic strain (Scherr et al., 2013; Zinoubi et al., 2018).

Although the results of this study indicate that the self‐paced stair ascent test could provide insight into aerobic capacity, there are some limitations to consider. Given the sample of 28 middle‐aged and older adults, consisting primarily of sedentary individuals, the generalizability of these findings to the broader adult population with more diverse physical activity and fitness levels is limited. Furthermore, despite the well‐established influence of confounders such as age and sex on functional capacity, the modest sample size in this study limits the reliability and stability of estimates and the accurate estimation of predictive performance in models with multiple predictors. Therefore, further studies are warranted to gain a deeper understanding of the feasibility of self‐paced stair ascent for assessing functional capacity.

## 5 CONCLUSIONS

This pilot study provides preliminary evidence that a self‐paced stair ascent test may be feasible field‐based approach for assessing aerobic capacity in middle‐aged and older adults. In particular, ascent time and mean vertical power were associated with both aerobic capacity and lower‐limb strength, suggesting that these simple measures may provide some insight into functional capacity. Additionally, incorporating information on perceived and relative physiological intensity during the ascent may further enhance the assessment of aerobic capacity, but appears less informative for lower‐limb strength. However, given the small sample size, these findings should be interpreted cautiously, and future studies with larger samples are needed to confirm the observations and to develop robust prediction models that also include relevant participant characteristics.

## CONFLICT OF INTEREST STATEMENT

The authors declare no conflicts of interest.

## Supporting information

Supporting File

## Data Availability

As applicable, the metadata of the study and anonymized research data will be available in JYX, the digital repository of the University of Jyväskylä with a DOI for permanent accessibility.
