# Supplementary material for: Feasibility of a field‐based submaximal stair ascent test for estimating aerobic capacity and lower‐limb strength in middle‐aged and older adults
Source: Clin Physiol Funct Imaging. 2026 Jun 5;46(4):e70073. doi: 10.1111/cpf.70073 (PMC13238290; doi:10.1111/cpf.70073)
Supplement: Supplementary file 1 — Supporting File [file CPF-46-0-s001.docx]

| **Four-flight stair ascent mean vertical power and RPE as predictors** | | | | |
| --- | --- | --- | --- | --- |
|  | **MODEL 1**  R^2^: 0.36  Adjusted R^2^: 0.31  MAE: 4.16 ± 3.43 | | **MODEL 2**  R^2^: 0.67  Adjusted R^2^: 0.62  MAE: 3.11 ± 2.28 | |
|  | B | 95% CI | B | 95% CI |
| Intercept | 22.66*** | (9.72, 35,61) | 48.46*** | (32.70, 64.21) |
| Mean vertical power [10W] | 0.69** | (0.31, 1.07) | 0.01 | (-0.43, 0.45) |
| RPE | -0.27 | (-1.17, 0.62) | 0.07 | (-0.62, 0.77) |
| Age [y] | n.a. |  | -0.17 | (-0.35, 0.02) |
| Sex (male = 0, female = 1) | n.a. |  | -10.55** | (-16.50, -4.60) |
| **Four-flight stair ascent mean vertical power and relative heart rate as predictors** | | | | |
|  | **MODEL 1**  R^2^: 0.36  Adjusted R^2^: 0.31  MAE: 4.33 ± 3.20 | | **MODEL 2**  R^2^: 0.67  Adjusted R^2^: 0.62  MAE: 3.10 ± 2.30 | |
|  | B | 95% CI | B | 95% CI |
| Intercept | 25.93*** | (3.43, 48.43) | 49.40*** | (29.05, 69.74) |
| Mean vertical power [10W] | 0.66** | (0.27, 1.06) | 0.02 | (-0.41, 0.45) |
| Relative heart rate [10%] | -0.74 | (-3.17, 1.69) | -0.04 | (-1.89, 1.81) |
| Age [y] | n.a. |  | -0.17 | (-0.35, 0.02) |
| Sex (male = 0, female = 1) | n.a. |  | -10.36** | (-16.17, -4.55) |
| **Six-flight stair ascent mean vertical power and RPE as predictors** | | | | |
|  | **MODEL 1**  R^2^: 0.45  Adjusted R^2^: 0.41  MAE: 3.86 ± 3.20 | | **MODEL 2**  R^2^: 0.68  Adjusted R^2^: 0.62  MAE: 3.11 ± 2.25 | |
|  | B | 95% CI | B | 95% CI |
| Intercept | 37.40*** | (19.92, 54.88) | 50.94*** | (34.85, 67.03) |
| Mean vertical power [10W] | 0.63*** | (0.30, 0.98) | 0.04 | (-0.42, 0.49) |
| RPE | -1.10* | (-2.18, -0.02) | -0.20 | (-1.18, 0.79) |
| Age [y] | n.a. |  | -0.16 | (-0.35, 0.02) |
| Sex (male = 0, female = 1) | n.a |  | -9.95** | (-16.50, -3.41) |
| **Six-flight stair ascent mean vertical power and RPE as predictors** | | | | |
|  | **MODEL 1**  R^2^: 0.38  Adjusted R^2^: 0.33  MAE: 4.30 ± 3.13 | | **MODEL 2**  R^2^: 0.68  Adjusted R^2^: 0.62  MAE: 4.30 ± 3.13 | |
|  | B | 95% CI | B | 95% CI |
| Intercept | 29.07** | (11.18, 46.95) | 51.52*** | (34.29, 68.76) |
| Mean vertical power [10W] | 0.66*** | (0.30, 1.03) | 0.02 | (-0.42, 0.46) |
| Relative heart rate [10%] | -0.99 | (-2.99, 1.00) | -0.33 | (-1.87, 1.21) |
| Age [y] | n.a |  | -0.17 | (-0.35, 0.02) |
| Sex (male = 0, female = 1) | n.a. |  | -10.26** | (-16.36, -4.16) |

**SUPPLEMENTARY TABLES**

**Supplementary table 1**. Apparent performance of the hierarchical regression models and their unstandardized regression coefficients in estimating VO_2_max normalized to body mass [ml·min^-1·^kg^-1^].

Model 1 includes best-performing pair of stair ascent predictors. Model 2 is additionally adjusted for age and sex. VO_2_max, maximal oxygen consumption; R^2^, coefficient of determination; MAE, Mean absolute error; CI, confidence interval. *** p < 0.001, ** p < 0.01, * p < 0.05

**Supplementary table 2**. Apparent performance of the hierarchical regression models and their unstandardized regression coefficients in estimating allometrically normalized VO_2_max [ml·min^-1·^kg^-2/3^].

| **Four-flight stair ascent mean vertical power and RPE as predictors** | | | | |
| --- | --- | --- | --- | --- |
|  | **MODEL 1**  R^2^: 0.54  Adjusted R^2^: 0.50  MAE: 16.66 ± 13.54 | | **MODEL 2**  R^2^: 0.79  Adjusted R^2^: 0.75  MAE: 11.65 ± 8.49 | |
|  | B | 95% CI | B | 95% CI |
| Intercept | 77.68** | (26.13, 129,23) | 187.26*** | (128.34, 246.19) |
| Mean vertical power [10W] | 3.92*** | (2.40, 5.44) | 1.06 | (-0.59, 2.72) |
| RPE | -1.28 | (-4.85, 2.30) | 0.17 | (-2.44, 2.78) |
| Age [y] | n.a. |  | -0.73* | (-1.43, -0.03) |
| Sex (male = 0, female = 1) | n.a. |  | -44.04*** | (-66.29, -21.79) |
| **Four-flight stair ascent mean vertical power and relative heart rate as predictors** | | | | |
|  | **MODEL 1**  R^2^: 0.53  Adjusted R^2^: 0.50  MAE: 17.21 ± 12.91 | | **MODEL 2**  R^2^: 0.79  Adjusted R^2^: 0.75  MAE: 11.64 ± 8.50 | |
|  | B | 95% CI | B | 95% CI |
| Intercept | 88.85 | (-1.01, 178.71) | 188.96*** | (112.92, 265.00) |
| Mean vertical power [10W] | 3.80*** | (2.23, 5.38) | 1.08 | (-0.54, 2.71) |
| Relative heart rate [10%] | -2.98 | (-12.69, 6.72) | -0.04 | (-6.95, 6.88) |
| Age [y] | n.a. |  | -0.73* | (-1.43, -0.03) |
| Sex (male = 0, female = 1) | n.a. |  | -43.63*** | (-65.35, -21.90) |
| **Six-flight stair ascent mean vertical power and RPE as predictors** | | | | |
|  | **MODEL 1**  R^2^: 0.60  Adjusted R^2^: 0.57  MAE: 14.92 ± 13.31 | | **MODEL 2**  R^2^: 0.79  Adjusted R^2^: 0.75  MAE: 11.97 ± 8.10 | |
|  | B | 95% CI | B | 95% CI |
| Intercept | 141.09*** | (71.36, 210.82) | 200.20*** | (139.67, 260.72) |
| Mean vertical power [10W] | 3.64*** | (2.27, 5.01) | 1.10 | (-0.62, 2.81) |
| RPE | -4.66* | (-8.98, -0.35) | -0.78 | (-4.50, 2.94) |
| Age [y] | n.a. |  | -0.74* | (-1.45, -0.02) |
| Sex (male = 0, female = 1) | n.a |  | -41.63** | (-66.26, -17.00) |
| **Six-flight stair ascent mean vertical power and RPE as predictors** | | | | |
|  | **MODEL 1**  R^2^: 0.54  Adjusted R^2^: 0.50  MAE: 17.09 ± 12.87 | | **MODEL 2**  R^2^: 0.79  Adjusted R^2^: 0.75  MAE: 11.62 ± 8.61 | |
|  | B | 95% CI | B | 95% CI |
| Intercept | 104.90** | (32.95, 176.84) | 202.07*** | (137.22, 266.92) |
| Mean vertical power [10W] | 3.74*** | (2.27, 5.20) | 1.03 | (-0.62, 2.68) |
| Relative heart rate [10%] | -4.08 | (-12.12, 3.95) | -1.25 | (-7.05, 4.55) |
| Age [y] | n.a |  | -0.76* | (-1.46, -0.05) |
| Sex (male = 0, female = 1) | n.a. |  | -42.88*** | (-65.82, -19.94) |

Model 1 includes best-performing pair of stair ascent predictors. Model 2 is additionally adjusted for age and sex. VO_2_max, maximal oxygen consumption; R^2^, coefficient of determination; MAE, Mean absolute error; CI, confidence interval. *** p < 0.001, ** p < 0.01, * p < 0.05

**Supplementary table 3**. Apparent performance of the hierarchical regression models and their unstandardized regression coefficients in estimating isometric leg press normalized to body mass [N·kg^-1^].

| **Four-flight stair ascent mean vertical power and RPE as predictors** | | | | |
| --- | --- | --- | --- | --- |
|  | **MODEL 1**  R^2^: 0.24  Adjusted R^2^: 0.18  MAE: 6.53 ± 4.76 | | **MODEL 2**  R^2^: 0.48  Adjusted R^2^: 0.38  MAE: 5.30 ± 4.16 | |
|  | B | 95% CI | B | 95% CI |
| Intercept | 32.56** | (13.15, 51,97) | 59.92*** | (32.40, 87.43) |
| Mean vertical power [10W] | 0.66* | (0.08, 1.23) | 0.58 | (-0.19, 1.35) |
| RPE | -1.01 | (-2.36, 0.33) | -1.04 | (-2.26, 0.18) |
| Age [y] | n.a. |  | -0.50** | (-0.82, -0.17) |
| Sex (male = 0, female = 1) | n.a. |  | 1.88 | (-8.51, 12.27) |
| **Four-flight stair ascent mean vertical power and relative heart rate as predictors** | | | | |
|  | **MODEL 1**  R^2^: 0.23  Adjusted R^2^: 0.17  MAE: 6.97 ± 4.19 | | **MODEL 2**  R^2^: 0.45  Adjusted R^2^: 0.35  MAE: 5.35 ± 4.36 | |
|  | B | 95% CI | B | 95% CI |
| Intercept | 42.55* | (8.53, 76.57) | 68.05*** | (31.69, 104.41) |
| Mean vertical power [10W] | 0.56 | (-0.03, 1.16) | 0.43 | (-0.35, 1.21) |
| Relative heart rate [10%] | -2.50 | (-6.17, 1.18) | -2.20 | (-5.50, 1.11) |
| Age [y] | n.a. |  | -0.47** | (-0.81, -0.14) |
| Sex (male = 0, female = 1) | n.a. |  | 0.60 | (-9.79, 10.99) |
| **Six-flight stair ascent mean vertical power and RPE as predictors** | | | | |
|  | **MODEL 1**  R^2^: 0.30  Adjusted R^2^: 0.24  MAE: 6.23 ± 4.70 | | **MODEL 2**  R^2^: 0.50  Adjusted R^2^: 0.41  MAE: 5.12 ± 4.14 | |
|  | B | 95% CI | B | 95% CI |
| Intercept | 41.29** | (14.05, 68.53) | 55.11*** | (27.55, 82.67) |
| Mean vertical power [10W] | 0.68* | (0.15, 1.22) | 0.88* | (0.10, 1.66) |
| RPE | -1.37 | (-3.06, 0.32) | -1.07 | (-2.77, 0.62) |
| Age [y] | n.a. |  | -0.48** | (-0.81, -0.15) |
| Sex (male = 0, female = 1) | n.a |  | 5.69 | (-5.53, 16.90) |
| **Six-flight stair ascent mean vertical power and RPE as predictors** | | | | |
|  | **MODEL 1**  R^2^: 0.29  Adjusted R^2^: 0.24  MAE: 6.44 ± 4.40 | | **MODEL 2**  R^2^: 0.52  Adjusted R^2^: 0.43  MAE: 4.63 ± 4.52 | |
|  | B | 95% CI | B | 95% CI |
| Intercept | 40.26** | (14.00, 66.52) | 59.98*** | (31.02, 88.93) |
| Mean vertical power [10W] | 0.70* | (0.17, 1.24) | 0.80* | (0.07, 1.54) |
| Relative heart rate [10%] | -2.36 | (-5.30, 0.57) | -2.06 | (-4.65, 0.52) |
| Age [y] | n.a |  | -0.50** | (-0.81, -0.18) |
| Sex (male = 0, female = 1) | n.a. |  | -4.17 | (-6.07, 14.42) |

Model 1 includes best-performing pair of stair ascent predictors. Model 2 is additionally adjusted for age and sex. R^2^, coefficient of determination; MAE, Mean absolute error; CI, confidence interval. *** p < 0.001, ** p < 0.01, * p < 0.05
